# Interaction with Industrial Digital Twin Using Neuro-Symbolic Reasoning

**DOI:** 10.3390/s23031729

**Published:** 2023-02-03

**Authors:** Aziz Siyaev, Dilmurod Valiev, Geun-Sik Jo

**Affiliations:** 1 Artificial Intelligence Laboratory, Department of Electrical and Computer Engineering, Inha University, Incheon 22212, Republic of Korea; 2 Augmented Knowledge Corp., Inha Dream Center, 100 Inha-ro, Michuhol-gu, Incheon 22212, Republic of Korea

**Keywords:** neuro-symbolic AI, artificial intelligence, digital twin, language understanding, internet of things, Industry 4.0, aircraft maintenance education, smart maintenance, Boeing 737

## Abstract

Digital twins have revolutionized manufacturing and maintenance, allowing us to interact with virtual yet realistic representations of the physical world in simulations to identify potential problems or opportunities for improvement. However, traditional digital twins do not have the ability to communicate with humans using natural language, which limits their potential usefulness. Although conventional natural language processing methods have proven to be effective in solving certain tasks, neuro-symbolic AI offers a new approach that leads to more robust and versatile solutions. In this paper, we propose neuro-symbolic reasoning (NSR)—a fundamental method for interacting with 3D digital twins using natural language. The method understands user requests and contexts to manipulate 3D components of digital twins and is able to read maintenance manuals and implement installations and removal procedures autonomously. A practical neuro-symbolic dataset of machine-understandable manuals, 3D models, and user queries is collected to train the neuro-symbolic reasoning interaction mechanism. The evaluation demonstrates that NSR can execute user commands accurately, achieving 96.2% accuracy on test data. The proposed method has industrial importance since it provides the technology to perform maintenance procedures, request information from manuals, and serve as a tool to interact with complex virtual machinery using natural language.

## 1. Introduction

Digital twins play a significant role in the industrial sector. Being a virtual representation of systems in the form of visualizations, 3D models, or virtual environments, digital twins provide a range of benefits to organizations, including improved design, effective decision making, and enhanced maintenance and operation of systems and products [1,2,3]. By simulating and analyzing complex systems, digital twins can provide new insights into their operation and maintenance and improve the quality and efficiency of applications for Industry 4.0 [4]. Numerous works have applied digital twins, for example, in the creation of the digital twin of a city as a foundation for analyzing the dynamics of the city in terms of processes and events [5], for a better understanding of urban drainage networks [6], and in space exploration satellites to check the operability of modules [7]. For this work, we used the most sophisticated example of a digital twin application in the aircraft maintenance industry since aircraft is one of the most complex machines that consist of millions of parts and complex assemblies [8,9]. Undoubtedly, digital twin technology innovates industrial applications; therefore, having effective interaction with digital twins is vital for successful use. Yet, digital twin technologies lack the ability to communicate with users in natural language, which reduces their potential effectiveness.

Natural language processing (NLP) is a rapidly growing field that has many practical applications in industries. Montejo-Ráez et al. [10] focused on emerging techniques and trendy applications of NLP, including text classification, text summarization, question answering, and machine translation. The use of attention models to solve question difficulty estimation in question-answering tasks, methods for generating more question-answer pairs in QA models, and a privacy-preserving machine reading system using transformer models and federated learning technologies are discussed. Massaro et al. [11] proposed a smart virtual front-office model that uses a combination of FAQs and a chatbot for industrial applications, with features such as self-learning, dynamic knowledge base generation, and improved user feedback. Mah et al. [12] highlighted the use of NLP and AI in enterprise management for Industry 4.0 to understand customer demands and improve customer satisfaction. Massaro et al. [13] described an acoustic training model for recognizing speech disorders, with real-time automatic scoring and a graphical dashboard for clinical evaluations and reporting. Alexakis et al. [14] introduced an IoT agent, a web application for monitoring and controlling a smart home remotely using NLP for user-friendly control of devices through text or voice commands. Although traditional NLP methods have proven to be effective in solving certain tasks, neuro-symbolic AI offers a new approach that leads to more robust and versatile solutions for NLP challenges.

Neuro-symbolic AI is a valuable technology for digital twins since it can understand complex structures and reason based on certain contextual knowledge, making it beneficial for human language understanding in digital twin contexts. Neuro-symbolic AI combines the strengths of both neural networks and symbolic reasoning, which helps complex systems to incorporate domain knowledge and perform complex reasoning and decision making based on that knowledge [15,16]. By extracting features using deep learning techniques, neuro-symbolic AI can understand various patterns, whereas specifically defined symbolic rules drive reasoning based on knowledge [17]. Neuro-symbolic AI has been applied in industries such as robotics, autonomous vehicles, and virtual assistants [18].

**Figure 1:**
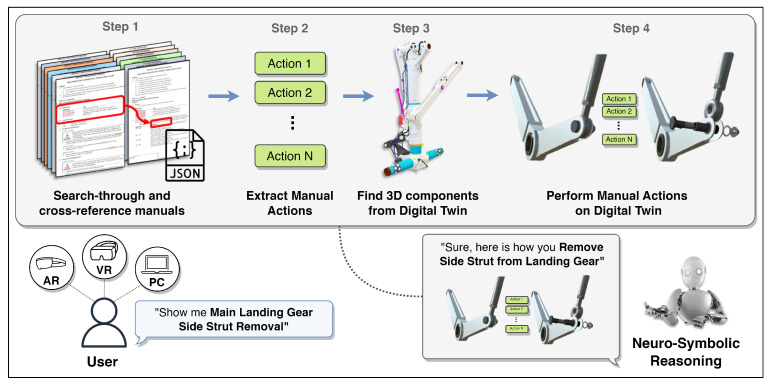
Neuro-symbolic reasoning for interaction with digital twins.

In this work, neuro-symbolic reasoning—a fundamental method for interacting with digital twins using natural language—is introduced (see Figure 1). The proposed method allows users to operate digital twins, perform installation and removal procedures, control 3D components, and request information from digital twin manuals. Users can communicate with digital twins using smart glasses or a PC in virtual environments and request procedures to be demonstrated. NSR can then understand the given query, search reference manuals to find actions to be performed, identify the required 3D components of the digital twin, and demonstrate visual animation of the requested execution. The digital twin interaction method consists of a neural translator, which is a machine translation model that converts user requests into symbolic programs, and a symbolic executor, which executes generated programs with respect to the user context. Along with this method, software for interacting with the digital twin and its resources is developed, where neuro-symbolic reasoning can demonstrate installation and disassembly procedures autonomously by following maintenance manuals.

**Figure 2:**
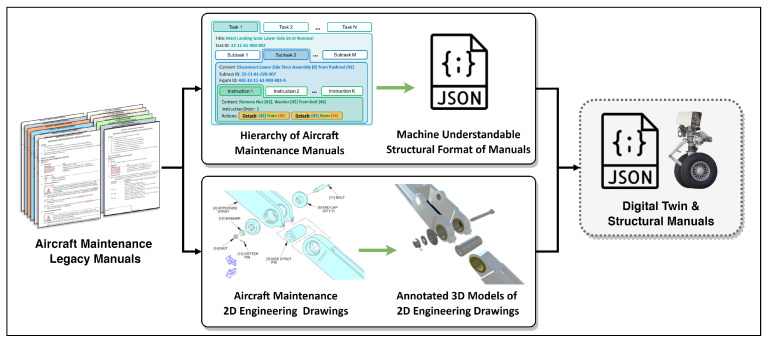
Digital twin content creation.

**Figure 3:**
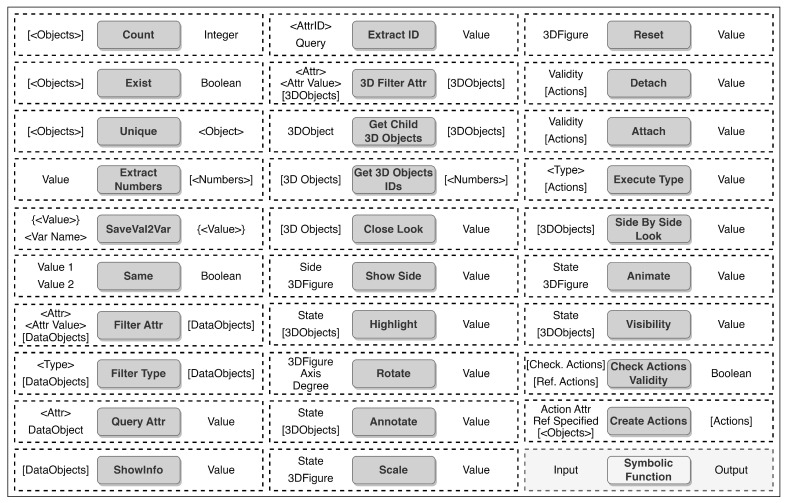
Digital twin interaction symbolic vocabulary.

A neuro-symbolic dataset for the digital twin is proposed to train NSR. Since the aircraft maintenance process is chosen as the use case for this work, we analyzed the legacy manuals of Boeing 737 [19] to convert them into a machine-readable format, keeping the standardized maintenance flow. Moreover, the 2D engineering figures present in the manuals are converted into annotated 3D models, which are cross-referenced with the created documents (see Figure 2). Once the annotated 3D digital twin is built, we design special symbolic vocabulary to represent the logic, functionalities, and decision-making process of neuro-symbolic reasoning (see Figure 3), where user commands are described with properly arranged sequences of symbols (symbolic programs). Furthermore, a dataset of queries is collected that includes possible user requests in a maintenance context, the corresponding symbolic programs, and ground-truth replies expected to be given by the system. Overall, the neuro-symbolic dataset consists of command-to-action and manual-based information retrieval queries for digital twin interactions.

The evaluation of the proposed neuro-symbolic reasoning is conducted using several machine translation and accuracy metrics on the test data. For the neural aspect of the method, various sequence-to-sequence neural networks are built and trained using training samples from the neuro-symbolic dataset of queries, mapping user requests to the corresponding symbolic programs. Next, the generated symbolic programs are executed to obtain the final responses, which are compared with the ground-truth replies. Overall, the experiments showed that machine translation models are able to translate the test examples with high accuracy, which indicates that the constructed symbolic logic in the dataset is consistent and well structured. NSR with a multi-layered GRU architecture as the backbone for the neural translator achieved the best results, with 96.2% neuro-symbolic accuracy, a BLEU score of 0.989, a BERTScore of 0.867, a ROUGE score of 0.994, and only a 0.2% failure rate. The evaluation demonstrated that NSR can understand new user requests and contexts and execute operations on the digital twin with high accuracy and a low failure rate.

The proposed neuro-symbolic reasoning has industrial value since it provides a methodology to perform the installation and removal of components in a digital twin, allows users to request information from maintenance manuals, and serves as a tool to interact and communicate with complex digital twins using natural language. Moreover, NSR can be adapted to create customized natural language interaction mechanisms for specific needs, addressing the high demand for the mass personalization of digital twins in Industry 4.0 [20]. In addition, the proposed method supports the rise of Industry 5.0, which is expected to focus on a more human-centered approach, and, in the context of human language understanding, will lead to more adaptable, dynamic, and personalized models that focus on understanding contexts to provide better and more human-like communication with digital twins [21].

In the following sections, the background knowledge about digital twins and the benefits of neuro-symbolic AI are discussed. Then, the creation of the neuro-symbolic dataset for the digital twin is explained, and in Section 4, the proposed neuro-symbolic reasoning is discussed in detail. The evaluation of the method is provided in Section 5. Finally, we present the results, contributions, and further research plans of this work in Section 6.

## 2. Background

### 2.1. Digital Twins

A digital twin [22,23,24] is a virtual replica of a physical object, device, or system that represents all functional features and data exchanges inside the components. A certain physical device is installed with different sensors for gathering data about vital areas of the environment. These sensors produce data on different important aspects of the physical system’s performance such as the energy output, temperature, humidity, etc. [25]. The data continuously flows between the existing physical and digital twins and they are strongly interconnected. Any changes made to the physical device automatically lead to an immediate update of its digital object and vice versa. Based on such direct and strong communication, the virtual replicas can run various simulations to study performance issues and generate possible improvement strategies, as well as provide valuable insights, which can be applied to the original physical object.

Digital twins are widely used in manufacturing, engineering, and other industries [1,2,3,4] to improve the design and performance of complex systems and allow simulations, which are hard to recreate in the real world under normal conditions. For example, Dimitrov et al. [5] created a digital twin of Sofia city as a foundation for the development of digital twin cities, representing the landscapes and urban areas, as well as the dynamics of the city, in terms of processes and events with high accuracy. Faced with persistent flooding and water quality challenges, water managers have developed digital twins of surface water systems that combine sensor data with online models to better understand and control system dynamics in natural/urban drainage networks [6]. Another proposed digital twin reference architecture in [26] has enabled the development of new applications as a service across the entire value life cycle to create interactions between people, smart devices, and wetlands. In addition, digital twins were developed for space exploration satellites in [7], where a platform to demonstrate the operability of modular satellites was simulated. In order to improve product quality and production efficiency, in [27], a system for a welding production line based on the physical production line of an equipment factory was built using digital twin technology. Furthermore, a basic model of a digital twin for the manufacturing environment of a micro-manufacturing unit was built, permitting the immediate integration of the machine into an industrial context and allowing the control of the parameters of the production system [28]. Chen et al. [29] proposed a framework in which behavioral models of drivers are shared among connected cars to predict the potential future actions of neighboring vehicles, thereby improving the safety of driving. Digital twins have also been introduced into architecture [30] to develop a procedure for creating an accurate virtual model, which integrates experimental physical reality and uses it to study the structural response of the system, its preventive maintenance, and strengthening operations.

In this work, an aircraft maintenance digital twin is applied as a use case for building neuro-symbolic reasoning for interactions. Digital twins in the aircraft industry have been implemented as the inner processes become increasingly digital, and the Internet of Things (IoT) has become increasingly dominant [4,31]. Digital twins can be used to model complicated structures and processes that uniquely interact with the surrounding environment, where one of the major challenges is the prediction of terminal effects over the entire life cycle of an aircraft [32]. The airframe, engines, flight control surface, instrumentation, pressurization, hydraulics, chassis, and other systems comprise the complicated aircraft’s intricate system architecture [8,9]. However, an aircraft collects a large amount of data during operation, which allows for the digital management of the aircraft’s full life cycle to minimize operating costs and increase system reliability.

Various works have applied aircraft digital twins to solve certain issues. To address the shortcomings of conventional approaches for certification, fleet management, and sustainment, the digital twin in [33] integrated ultra-high-fidelity simulation into an aircraft’s onboard health management system, maintenance history, and available historical and fleet data to mirror the life of its flying twin and enable unprecedented levels of safety and reliability. Additionally, “virtual reality + Internet” were combined in an experimental power supply system for large multi-electric aircraft [34], resulting in the development of a virtual simulation platform. This system was created for the power supply management and fault reconstruction of multi-electric aircraft and facilitates the sharing of industrial and social resources. Oyekan et al. [35] proposed an industrial robot with a computer vision system and a digital twin that is used to create an automation cell for the fan-blade reconditioning component of aerospace maintenance, repair, and overhaul (MRO) services, as well as the digitization of the fan-blade surface, tracking, and guidance of material removal. Furthermore, virtual assembly was presented in [36], which realizes the assembly process design, verification, and optimization of complex products in the virtual environment. This system has been effectively used to improve the assembly quality and efficiency of complex products.

In order to build an interaction mechanism with digital twins, the neuro-symbolic AI concept is applied, which demonstrates great potential for the problems mentioned above. Therefore, in the next section, neuro-symbolic AI and its methodology are introduced.

### 2.2. Neuro-Symbolic AI

An emerging AI domain that shows potential for use in digital twin technology is neuro-symbolic AI (NSAI) [15,16], which blends deep learning for feature extraction with rules-based “intuition” for manipulating those features. Until the 1980s [37], rule-based or symbolic techniques dominated the field of artificial intelligence. Compared to deep learning models, symbolic models require fewer input samples, can be successfully extended to new problems, and their core functionality is conceptually straightforward. At the same time, they require a significant amount of hand-tuning, making it difficult to develop complex issues. Therefore, neuro-symbolic AI [16,37,38,39] was not a major concern until recently, when key advances in machine learning driven by neural networks, led to an enormous increase in interest and research work on integrating neural and symbolic approaches. NSAI [17] is a combination of neural networks and symbolic techniques that aims to take advantage of the strengths of each. Deep learning has demonstrated exceptional performance in extracting complicated features from data in applications such as object detection and natural language processing. Simultaneously, symbolic AI is useful for formalizing human-like reasoning. The goal of NSAI [40] is to extract features from data using deep learning techniques and then utilize these features using symbolic techniques.

NSAI [18] has been used to improve systems that now use AI such as robotics, autonomous vehicles, or digital assistants. The project in [41] used neuro-symbolic AI to create methods that can make transparent predictions in the context of drug repurposing and the authors aimed to understand how the organization of data in a knowledge graph changes the quality of predictions. Bennetot et al. [42] presented a probabilistic programmed deep-kernel learning approach to the personalized, predictive modeling of neurodegenerative diseases while considering a spectrum of neural and symbolic machine learning approaches, which assess predictive performance and important medical AI properties such as interpretability, uncertainty reasoning, data efficiency, and domain knowledge. Moreover, Morel et al. [43] advocated a merging of two trends—neuro-symbolic AI and the smart city—and worked toward the complete integration of these two technologies. In addition, [44] described a platform and research efforts that approximate the elements that underlie human cognition based on the physical embodiment and environmental context, which is achieved by combining human-like robotic grasping and social abilities with symbolic AI with explicit knowledge models and inference and deep learning networks all within in an adaptive, reconfigurable framework.

Therefore, the concept of neuro-symbolic AI to build NSR for interaction with digital twins is applied, where a question-and-answer system is created to communicate with the digital replica to find a practical solution to the industrial problem.

## 3. Digital Twin and Neuro-Symbolic Dataset

### 3.1. Overview

This work starts with building the digital twin and datasets for creating the interaction mechanism. To develop a dataset for an aircraft maintenance digital twin, extensive work with legacy manuals is carried out since they provide procedures that need to be strictly followed. The manuals cross-reference each other, providing maintenance steps and related items to work with. Therefore, to create the digital twin of aircraft maintenance, the following steps are performed, as described in Figure 2.

First, a hierarchy of existing aircraft maintenance manuals is identified to convert them into a machine-readable format called JSON. All maintenance manuals and operations are divided into sections (ex. landing gear). A section has tasks (installation or removal of an aircraft part), a task has subtasks (procedures that concentrate on certain assemblies of that part), and a subtask has instructions (detailed operations to be performed on items). See Figure 2 for a detailed example.

Second, 2D engineering drawings are converted into annotated 3D models. There are numerous figures presented in the manuals that show components, relationships, and annotations. We recreated the manuals in a 3D format, building the components of the digital twin using the annotations from the legacy manuals. An annotation contains information about an item, its installation or removal configuration, and many other features.

Combining the assets from previous steps, the digital twin metadata is obtained, which consists of machine-readable manuals and 3D models that are interconnected. Overall, 5 tasks, 32 subtasks, and 100 instructions are built, and 3D models of each of the maintenance operations are created. Next, to give the digital twin assets operability and develop the reasoning mechanism, the neuro-symbolic dataset is introduced.

### 3.2. Neuro-Symbolic Dataset

The underlying principle of users’ interactions with digital twins is depicted in the neuro-symbolic dataset, which consists of user requests and their translations into machine language. The first step in building the dataset is to create a symbolic vocabulary that describes procedures or operations that can be applied to the digital twin. The second step is to map the user requests to the symbols in the vocabulary that describe the translation. Therefore, a dataset of requests is created, which is provided in a specific context that translates user queries in English into symbolic programs that the system can understand, resulting in the completion of the requested operations. The dataset is used to train neuro-symbolic reasoning and evaluate the system and is described in the later sections.

#### 3.2.1. Symbolic Vocabulary

The proposed symbolic vocabulary represents operations that can be applied to the digital twin. The vocabulary consists of symbols that describe the digital twin’s functions and their possible configurations. Figure 3 illustrates the symbolic vocabulary built to interact with the aircraft maintenance digital twin. Overall, there are 30 operations, each with a specific purpose. The symbolic vocabulary consists of terminal and non-terminal symbols. Terminal symbols represent a final operation to be performed on the digital twin. In contrast, non-terminal symbols are used to retrieve or compute information for the functional parameters of a terminal node. The following are definitions of some terminal symbols:

- Attach-Performing installation of object A on object B;
- Detach-Performing disassembly of object A from object B;
- Rotate-Performing rotation operation on object A on specific angle B.

The following are examples of non-terminal symbols:

- FilterType-Traversing information nodes and filtering of certain types of nodes;
- Count-Counting the number of objects in a given list;
- 3D Filter Attr-Traversing 3D components and filtering of models based on attributes and values.

Overall, the symbolic vocabulary aims to interact with the digital twin and request information from manuals. Once the symbolic vocabulary is created, a translation of simple English into the created symbolic language needs to be constructed. Therefore, in the next section, the creation of user query samples and the corresponding symbolic programs is explained.

#### 3.2.2. Neuro-Symbolic Dataset of Queries

**Figure 4:**
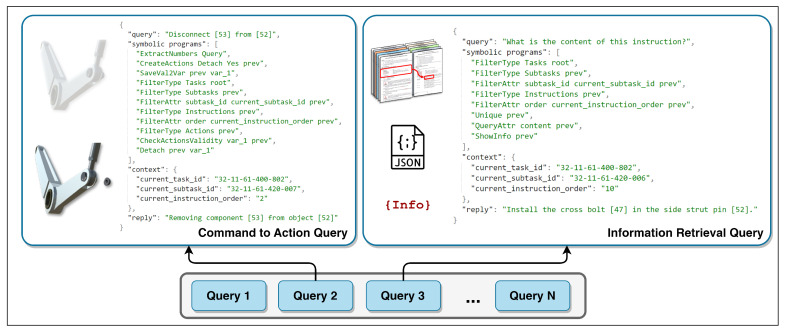
Neuro-symbolic dataset of queries.

To utilize the symbolic vocabulary, the dataset of user queries is proposed. The dataset consists of numerous examples of natural language requests that are translated into a series of symbolic programs. Considering that all the queries are taken from the aircraft manuals, the contexts of the requests alongside the queries are defined. Correspondingly, the expected ground-truth answer that should be provided by the system is also supplied for each sample. Figure 4 demonstrates an example of a query. The following are the definitions of the variables used in the queries:

- “query” represents the user requests;
- “symbolic programs” defines the corresponding symbolic programs;
- “context” provides information on the task, subtask, and instruction of the query;
- “reply” shows the expected answer from the execution of the request.

There are two types of requests in the dataset (see Figure 4). The first type is command-to-action requests. These requests are for performing an operation on the digital twin. The operation can be the installation or removal of certain parts or the manipulation of the 3D model such as rotation, scaling, etc. As can be seen from the example in Figure 4, the request “Disconnect [53] from [52]” results in uninstalling the item from the assembly.

The second type of query in the dataset is information retrieval requests, which compute or extract the requested information from the aircraft manuals discussed in Section 3.2. The execution of the programs in the query, such as “show the content of this instruction”, results in the retrieval of the answer from the document according to the given context in the manual.

Overall, there are 9000 queries created. The queries are made by people and automation software written in Python, altering the various needs, contexts, conditions, and semantics of the language. The average number of symbols per query is 23.47, with a standard deviation of 10.46, and an average of 6.14 symbolic programs are constructed with a standard deviation of 2.48. Taking the example in Figure 4, the first query with the request “Disconnect [53] from [52]” is converted into 11 programs, whereas, the second sample query with the request “What is the content of this instruction?” is represented by 8 symbolic programs. The proposed requests reflect the functionality of the developed system when interacting with the digital twin. Our neuro-symbolic dataset addresses real-world problems and can be applied in industries that require the use of digital twins and document manuals. The proposed dataset is applied to build the neuro-symbolic interaction mechanism introduced in the next section.

## 4. Neuro-Symbolic Reasoning

### 4.1. Overview

In this work, we propose neuro-symbolic reasoning to operate a digital twin and ask manual-related questions. Users are able to interact with the 3D assets of a digital twin and request that various operations be demonstrated by making queries in English. Moreover, special digital twin interaction software is developed that allows users to experience communication with an aircraft maintenance digital twin.

The proposed digital twin is created in 3D; therefore, it can be easily introduced into smart devices and virtual environments, including Augmented Reality (AR), Virtual Reality (VR), or Mixed Reality (MR), since the digital twin is represented in 3D. Moreover, with the help of NSR, a user wearing smart glasses or using a PC can query the system and receive a reply. Figure 1 illustrates the concept of the proposed reasoning. Once a user makes a request, NSR understands the query and searches and cross-references manuals, extracting relevant information for execution; it finds 3D assets of the digital twin mentioned in the maintenance documents and performs computed operations with visual feedback to the user. There are several steps involved in the proposed neuro-symbolic reasoning, which are described in the following sections.

### 4.2. Understanding User Requests

**Figure 5:**
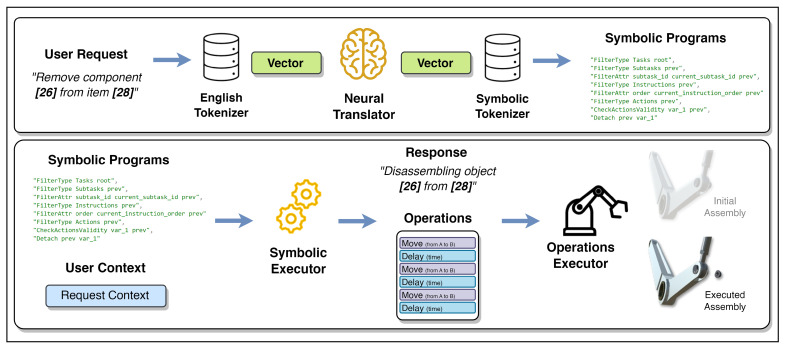
Neuro-symbolic reasoning pipeline.

The first stage in neuro-symbolic reasoning is understanding requests. This step can be considered machine translation since requests given in a human language need to be converted into machine-understandable symbols defined in the system. Using the neuro-symbolic dataset described in Section 3.2, a neural translator is developed, which parses the questions into a series of symbolic programs (see Figure 5).

The underlying technology for the neural translator is a sequence-to-sequence neural network that translates a sequence of English word tokens that represent requests into a sequence of tokens from the symbolic vocabulary, which reflect symbolic programs. The neural translator is trained on the neuro-symbolic dataset of queries presented in Section 3.2.2. Taking the “query” and “symbolic programs” data fields of the requests, the neural translator’s neural network is trained. The choice of a machine translation neural network can vary based on tasks and datasets; however, in our work, a multi-layered GRU architecture is chosen due to its superior performance over other architectures (see Section 5).

Figure 5 describes the inference flow. First, users’ queries are converted into vectors by the English language tokenizer. Second, the neural translator performs sequence-to-sequence translation, creating the symbolic vector. Lastly, the symbolic vocabulary tokenizer converts the generated vectors into symbolic programs, which are defined in the system (see Figure 3).

The task of the neural translator is to convert user requests into a series of symbols for the machine to execute. It tells the neuro-symbolic reasoning what steps it needs to perform to obtain the answer to the question. Therefore, once the system has computed the next steps that need to be taken, the symbolic executor of NSR takes over.

### 4.3. Execution of Operations

The symbolic executor is a reasoning mechanism that executes generated symbolic programs based on user context. Once the neural part of the reasoning provides symbolic programs, the symbolic executor performs step-by-step computations to create a response (see Figure 5).

The symbolic executor provides two types of feedback depending on the request. In Section 3.2, it was discussed that information retrieval and command-to-action queries exist in the dataset. For information retrieval queries, the symbolic executor provides results in a text format and summarizes the extracted information from the manuals, whereas for command-to-action requests, the symbolic executor generates primitive operations and performs actions on the 3D components of the digital twin (see Figure 5).

**Figure 6:**
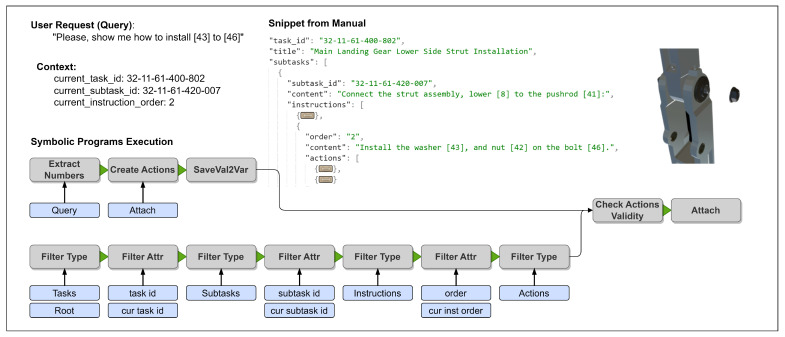
Example of execution of symbolic programs.

To better understand the execution of the symbolic programs, an example is provided in Figure 6. Assuming that the user request is “Please, show me how to install [43] to [46]”, which is a command-to-action query type, the neural translator generates the corresponding symbolic programs. In addition, the context of the request is provided, including information about the current task, its subtask, and the instruction order, which defines a specific manual process. A manual snippet of a given context is provided below.

The symbolic executor starts by executing one symbolic program at a time, passing the result from the execution to the next stage. The symbols in a program represent a function and the function parameters that need to be passed in the given order. Below is the reasoning mechanism of the example in Figure 6 described:

1. ExtractNumbers Query—Extract numbers from the query, where the query is a symbol that represents a user request in the system. The outputs of this step are the numbers that exist in the query.
2. CreateActions Attach—Based on predicted parameters, queries in the form of actions are represented in case the query is a command-to-action type.
3. SaveVal2Val—Saving computed values from the previous execution to be used later. Some queries are more complex than just sequential processing; therefore, the temporary values are stored.
4. FilterType Tasks Root—Traversing manuals to find nodes with type tasks.
5. FilterAttr task_id current_task_id—Traversing tasks to find the task of the context.
6. FilterType Subtasks—Traversing the current task to find nodes with type subtasks.
7. FilterAttr subtask_id current_subtask_id—Traversing subtasks to find the subtask of the context.
8. FilterType Instructions—Traversing the current subtask to find nodes with type instructions.
9. FilterAttr order current_instruction_order—Traversing instructions to find the instruction of the context.
10. FilterType Actions—Traversing the current instruction to find nodes with type actions.
11. CheckActionsValidity—Checking whether the requested actions from the user query, which are saved in the temporary variable in step 3, overlap with valid actions of the current context.
12. Attach—Performing actual actions requested by the user in case they are valid.

Command-to-action queries generate primitive operations if the validation of the request is confirmed. These primitive operations consist of a sequence of generic steps that need to be applied by the executor of the operations. In the example in Figure 5, it is shown that the removal action of one item from another is represented as primitive move and delay steps. For users, it is crucial to be able to see a proper visual response; therefore, the request’s execution must be plausible and understandable in terms of both the process and results.

All assets of the digital twin, neuro-symbolic queries, and the reasoning mechanism let users experience communication with the digital twin, which is accessible through special software built for this work, discussed next.

### 4.4. Digital Twin Interaction Software

**Figure 7:**
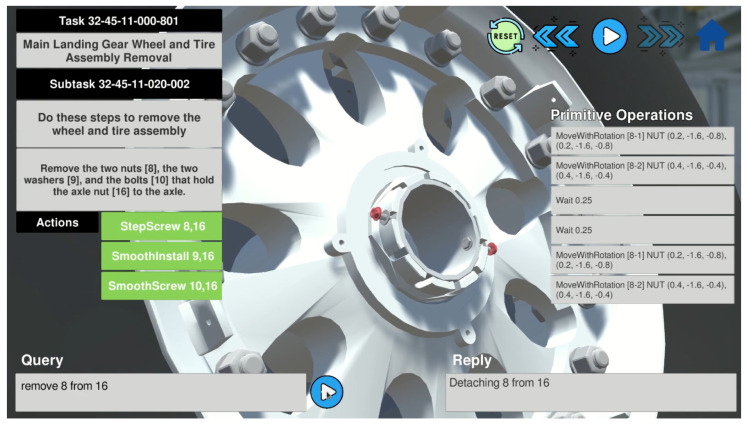
Digital twin interaction software.

Digital twin interaction software is developed to allow users to make requests and explore the digital twin and neuro-symbolic datasets (see Figure 7). Users can choose a certain context in the aircraft maintenance digital twin such as a task, a subtask, or an instruction. Next, the system loads all the resources and presents the 3D digital twin and its specific parts in the software. Users can choose whether to automatically execute the actions of the context or make personalized requests. The digital twin interaction software is developed in Unity [45], which is a cross-platform game engine that supports a variety of desktop, mobile, and virtual reality platforms. Unity can be used to create 3D and 2D games, as well as interactive simulations and other experiences.

The proposed neuro-symbolic reasoning is applied to each stage of the execution of the digital twin in the software. Symbolic programs are utilized to find manual nodes from the dataset, load the corresponding parts of the digital twin, and prepare actions for execution. In a case where users want to view procedures, the play button in the top right corner can be used to trigger a demonstration (see Figure 7).

The query field in the software allows users to make custom requests. Users type their requests in the text area and NSR is triggered to perform the inference described in Figure 2. Various operations can be performed with the aircraft maintenance digital twin, including its manipulation, exploration, and simulation. Since the neural translator is trained with variations of the requests, it is able to understand different semantics in the queries.

The interaction software incorporates communication protocol between the user and the digital twin, which is accessed as follows: wearing smart glasses or using a PC, the user requests are sent to a deep learning machine (server) that executes neural networks (the neural translator) and delivers the computed symbolic programs to a client device over the Internet.

The snapshot of the software in Figure 7 is a PC version; however, the digital twin and interaction mechanism can be built into AR, VR, and MR environments. The PC version allows for the comfortable use of a computer keyboard to type queries, whereas in the case of smart glasses, an integrated speech module can be utilized to convert spoken user requests into text and interact with the digital twin using NSR in the same way.

### 4.5. Choosing Neuro-Symbolic AI over Neural Networks

Applying neuro-symbolic AI in digital twins has several advantages over conventional neural networks. We identified the most relevant benefits of NSR with respect to our work and provide a summary below:

- Scalability. Digital twins usually represent complex structures that may consist of millions of components (for example, aircraft maintenance digital twins) that change regularly. Therefore, using neural networks with embedded context is not scalable since every time a new context appears, neural nets must be retrained to be updated. On the other hand, neuro-symbolic AI has a mechanism to interact with context (manuals and 3D models) instead of memorizing it. This makes the modifications of manuals and 3D models much easier, without the need to change the reasoning.
- Explainability. The reasoning mechanism of neural networks is a black box and the results obtained are not explainable. In contrast, in our neuro-symbolic AI, the execution of symbolic programs can be analyzed step by step, bringing clarity to the inference process, and understanding the intermediate outputs.
- Finer Modifications. Along with the explainability of neuro-symbolic AI, finer modifications to the reasoning mechanism are possible. Since the intermediate steps in the execution of symbolic programs can be analyzed, a certain symbol can be updated based on needs, reflecting changes in the system. Therefore, the neural part of neuro-symbolic AI can remain unchanged while modifying the symbolic AI only, which is not the case for neural networks since the only solution is to retrain them.

Overall, question-answer and interaction systems that rely on the context during inferences, such as interaction with digital twins for complex machinery, should apply neuro-symbolic reasoning to maintain the context and understand complex human requests. In the next section, the evaluation process for the proposed NSR is performed.

## 5. Evaluation

In this section, the evaluation of the proposed neuro-symbolic reasoning is described. First, the training and test data used for the experiments are presented. Then, the assessment metrics and evaluation strategy, along with the results, are discussed.

### 5.1. Data

The data for the evaluation are sampled from the neuro-symbolic dataset of queries and the digital twin created with manuals. As described in Section 3, the neuro-symbolic dataset of user requests, programs, contexts, and replies based on cross-referenced digital twin and aircraft manuals was collected. All queries were divided into two categories, that is, information retrieval and command-to-action types. Overall, about 9000 user requests provided in specific contexts, their corresponding symbolic programs, and expected ground-truth replies after the execution of the requests with NSR were obtained.

For evaluation purposes, two sets of data from the available queries in the dataset were randomly sampled:

- Training Data—8000 queries;
- Test Data—1000 queries.

All the requests from the neuro-symbolic dataset have unique characteristics, including request texts, digital twin and manual contexts, and corresponding replies. The evaluation process was performed using these two sets of data and is described in the next section.

### 5.2. Metrics and Strategy

The evaluation strategy included the assessment of the intermediate steps and a full inference pipeline of neuro-symbolic reasoning. We evaluated the proposed method with several neural network models, altering the neural translator of NSR. The networks were trained with the training data and using the evaluation metrics, the performances of all the setups were assessed on the test data.

First, various machine translation architectures were built for the neural component, the neural translator. State-of-the-art sequence-to-sequence neural network architectures were selected, including multi-layered LSTM, GRU, and transformer with attention mechanisms, to develop several versions of the neural translator. All models have encoder–decoder architecture that encoded the source sentence (user request) into a single vector. The request vector was then decoded by a decoder network, which learned to output the target sentences (symbolic programs). The encoder and decoder were made up of multiple layers of recurrent neural networks (RNNs) or transformer blocks. In the case of the RNNs, the layers were long short-term memory (LSTM) units or gated recurrent units (GRUs). For the transformer blocks, the layers were made with multi-head self-attention mechanisms and point-wise feed-forward neural networks. The following is a summary of the neural models built for the experiments:

- Multi-layered LSTM—multi-layered long short-term memory (LSTM) [46] from the following work [47].
- Multi-layered GRU—Multi-layered gated recurrent unit (GRU) [48]. A GRU is similar to LSTM but has two gates: the update gate and the reset gate [49].
- GRU with Attention—Multi-layered gated recurrent unit with decoder attention, which applies an attention mechanism by computing the weighted sums of the encoder’s hidden states [50].
- GRU with Padding—Multi-layered gated recurrent unit architecture that applies packed padded sequences and masking. Padded sequences force the model to skip padding tokens in the encoder, whereas masking allows the network to ignore attention over padded elements.
- Transformer Attention—Multi-layered transformer model with attention mechanisms [51]. The encoder and decoder are made of multiple layers, with each layer consisting of multi-head attention and position-wise feed-forward sublayers.

Second, these sequence-to-sequence models were trained with the training data, as described in the previous section. All the networks had the same training setup, including the training data, environment, etc. The models had encoding and decoding dimensions of 256 and hidden dimensions of 512. The dropout applied to all encoder and decoder models was 0.5. The training was performed using the Adam optimizer with a learning rate of 0.001, cross-entropy loss, and training batch size of 512 for all the network architectures.

Next, using machine translation and classification metrics, the performance of each setup was assessed on the test dataset, which was allocated for evaluation purposes only. The following evaluation metrics were applied:

- Neural accuracy is the ratio of the number of correctly predicted symbolic programs (or translated user requests) to the total number of input pairs (requests to programs).
- Bilingual Evaluation Understudy (BLEU) [52] is a metric used to assess automatic translation by measuring the difference between reference translations made by humans and machines for the same source sentence. The metric is language-independent and correlates highly with human evaluation. Since a narrow domain is applied in this work, a high BLEU score is expected [53].
- BERTScore [54] is a metric for the evaluation of automatic text generation. The BERTScore applies the pre-trained embeddings from the BERT model [55] and computes the semantic similarity between the reference and predicted tokens. The metric computes the cosine similarity between each word from the reference and predictions.
- Metric for the Evaluation of Translation with Explicit Ordering (METEOR) [56] is a machine translation evaluation technique that matches unigrams between the reference and predicted translations. METEOR is based on the harmonic mean of unigram precision and recall, giving higher weights to recall. It produces a substantial correlation with human judgment at the sentence level [57].
- Recall-Oriented Understudy for Gisting Evaluation (ROUGE) [58] is a measure that counts the number of overlapping units, such as n-grams, word sequences, and word pairs, between machine-generated predictions and human-produced references [58]. In our case, ROUGE-1 is applied, which compares the similarity of unigrams between the reference and predicted summaries.
- Neuro-symbolic accuracy is the ratio of the number of correctly predicted responses (or executed symbolic programs) based on a given query and context to the total number of examples. This metric represents the accuracy of the whole neuro-symbolic reasoning pipeline.
- Failure rate is the ratio of the number of unsuccessful executions of generated symbolic programs to the total number of symbolic programs.

Overall, five NSR setups were checked, altering the neural network model of the neural translator and evaluating the execution ability and correctness of the whole system. In the next section, the experimental results are presented.

### 5.3. Results and Discussion

In this section, the results and discussion of the evaluation of NSR are described. Using the test data and the various metrics presented in the previous sections, the experiments are summarized as follows: first, the neural translator is evaluated by assessing its ability to perform machine translations of user requests into symbolic programs (see Table 1); and second, the results of the assessment of overall neuro-symbolic reasoning (see Table 2) are shown.

As shown in Table 1, with regard to the accuracy of the neural translator, the best results were achieved by the multi-layered GRU and transformer models with 96.2% and 96% accuracy rates, respectively. This demonstrates that the mentioned sequence-to-sequence models are able to accurately translate unseen user requests from the test data into symbolic programs. As can be seen in Table 1, these models showed the best performance among the network architectures, with the results being nearly equal in all metrics.

The BLEU score of the assessment models ranged from 0.903 to 0.989 for multi-layered LSTM and multi-layered GRU, respectively. The BLEU scores were calculated for individual translated sentences (symbolic programs) by comparing them with a set of reference translations, and since the BLEU scores were high, this indicates that the predicted symbolic programs were similar to the ground-truth programs, with some slight differences or a different construction order.

It can be seen that the BERTScore for almost all the machine translation models was the same at 0.86. The BERTScore focuses on computing the semantic similarity between tokens using contextual embeddings, which may explain the above results, as our symbolic language vocabulary was very limited and the representations for certain meanings were unique. In the case of the METEOR and ROUGE metrics, which both compare the similarity of unigrams, the highest scores were achieved by the multi-layered GRU architecture with scores of 0.944 and 0.994 for METEOR and ROUGE, respectively.

Assessing NSR (Table 2), the results of neuro-symbolic accuracy were almost identical to neural accuracy. This shows that if symbolic programs are generated incorrectly, the output of symbolic execution, in most cases, is also wrong since NSR heavily depends on the neural translator. Overall, the multi-layered GRU and transformer models for the neural translator showed a 96% performance rate for NSR.

Comparing the GRU-based models, the results show that the more complex GRU networks, either with special decoder attention or packed padded sequences and masking techniques, were less effective compared to simple multi-layered GRU. GRU with attention and GRU with padding achieved 10.6% and 4.8% lower neuro-symbolic accuracy, respectively.

The greatest failure rate in the experiments was 1.6%, which was only 16 failed executions out of 1000 test samples. This demonstrates the excellent ability of the sequence-to-sequence models to construct symbolic programs with proper structural arrangements of functions and symbolic attributes and shows the capability of machine translation models to predict the next token in the sequence. Thus, even though the multi-layered LSTM architecture showed the worst performance among all the architectures with only 44% of neural and neuro-symbolic accuracy, it only had a 0.1% failure rate. In contrast, multi-layered GRU showed exceptional results in the failure rate metric, with only 0.2% of unsuccessful executions, which was the lowest among the architectures. One reason why the multi-layered LSTM model may have exhibited lower accuracy is a lack of data. LSTMs are complex models that require a large amount of data to effectively train. If the model is not trained on enough data, it may not be able to learn the underlying patterns in the data, resulting in poor performance on the test data. Similarly, if the problem is too complex for the current model’s architecture, more data may be needed to improve performance.

In summary, the evaluation results showed that the proposed neuro-symbolic dataset is consistent since the machine translation models were able to converge on the training data, achieving high accuracy on the test data. In addition, the results demonstrated that the multi-layered GRU and transformer architectures were the most suitable for use in the neural translator of NSR. Overall, 96.2% of neuro-symbolic accuracy was achieved on the test data, with only a 0.2% failure rate for symbolic programs. The ontology of neuro-symbolic reasoning is embedded in the manuals and digital twin, and when a user makes a request in natural language, the neural translator interprets the request, creating a series of instructions (symbolic programs) to be performed with the knowledge base. As the system trains, it learns to map user requests to symbols from the symbolic vocabulary. To provide a response, the symbolic executor executes the instructions step by step, accessing information from the ontology. Therefore, NSR is a scalable solution as it has a mechanism for interacting with the context, making modifications to manuals and 3D models much easier without the need to change reasoning.

## 6. Conclusions and Future Works

To conclude, digital twins have gained popularity in industrial applications since they can help to predict a system’s behavior and performance. As they are in a virtual environment, they can simulate and analyze the digital replica of a physical object or system to identify potential problems and opportunities for improvement. Furthermore, the ability to effectively interact with digital twins plays a crucial role in their successful use and natural language is the most convenient interaction mechanism. Recent advances in neuro-symbolic AI that combine both neural networks and symbolic reasoning to solve complex problems allow for the interpretation of the sophisticated structures of digital twins and their operation using natural language requests.

In this work, we proposed the neuro-symbolic reasoning mechanism—a fundamental way of interacting with digital twins using natural language. The proposed method allows users to perform the installation and removal of digital twin components, manipulate 3D models, and obtain specific knowledge from manuals using language dialog with the digital twin. To accomplish this, a neuro-symbolic dataset of the digital twin was created. First, the aircraft maintenance digital twin of a Boeing 737 was constructed by converting maintenance manuals into a structural machine-readable format and transforming 2D engineering drawings into annotated 3D models. Second, a special symbolic vocabulary was created, where symbols represent the components and functionality of the digital twin. Third, a neuro-symbolic dataset of queries was collected, consisting of user requests given in specific contexts and their corresponding symbolic programs. The proposed dataset has industrial importance since it demonstrates the use of neuro-symbolic AI in a real-world application. Moreover, NSR was proposed, which allows intuitive interaction with the digital twin through simple language communication. It consists of a neural translator, which understands user requests and translates them into symbolic programs, and a symbolic executor, which executes the symbolic programs considering the user context. In addition to natural language understanding, the system is able to read structured maintenance manuals and implement assembly and disassembly procedures autonomously.

An evaluation of NSR was conducted. By altering various sequence-to-sequence models for the neural translator, the system was trained using a training dataset and experiments were carried out using test data. The individual components and the overall reasoning mechanism were assessed using various machine translation and classification metrics. To sum up, the experimental results demonstrated that the proposed neuro-symbolic dataset is consistent and well structured since the machine translation models were able to translate the test examples with high accuracy. The best NSR setup was achieved with the multi-layered GRU neural translator, which demonstrated neuro-symbolic accuracy of 96.2%, a BLEU score of 0.989, a BERTScore of 0.867, a ROUGE score of 0.994, and only a 0.2% failure rate. This shows that NSR can understand new user requests and contexts and perform executions with high accuracy and a low failure rate.

To sum up, the contributions of this work include the following: a method for understanding natural language using neuro-symbolic reasoning for intuitive and explainable interactions with digital twins; a virtual aircraft maintenance digital twin with structured manuals; a neuro-symbolic dataset of user queries for operating the digital twin; and digital twin interaction software, which provides an environment for users to interact with the industrial digital twin.

In the future, it is planned to enhance neuro-symbolic reasoning to understand the shape, structure, and purpose of 3D components of digital twins using neural AI to further eliminate the need for metadata. This would require building neural models capable of predicting the relationships between 3D objects regardless of their features. We truly believe that NSR is a sustainable technology for digital twins that will bring numerous innovations to the field.

## Figures and Tables

**Table 1:** Neural translator evaluation.

Model	Accuracy	BLEU	BERTScore	METEOR	ROUGE
Multi-layered LSTM	0.446	0.903	0.866	0.927	0.956
**Multi-layered GRU**	**0.962**	**0.989**	**0.867**	**0.944**	**0.994**
GRU with Attention	0.856	0.965	0.866	0.935	0.980
GRU with Padding	0.907	0.974	0.866	0.938	0.986
Transformers Attention	0.960	0.986	0.867	0.942	0.993

**Table 2:** Neuro-symbolic reasoning evaluation.

Model	Neural Accuracy	Neuro-Symbolic Accuracy	Fail Rate
Multi-layered LSTM	0.446	0.449	0.010
**Multi-layered GRU**	**0.962**	**0.962**	**0.002**
GRU with Attention	0.856	0.856	0.010
GRU with Padding	0.907	0.914	0.016
Transformers Attention	0.960	0.960	0.006

## Data Availability

Data are available on request due to privacy restrictions.
